# *Rhizobium* Phage-Like Microvirus Genome Sequence Identified in Wastewater in Arizona, USA, in November 2020 Encodes an Endolysin and a Putative Multiheme Cytochrome *c*-like Protein

**DOI:** 10.1128/mra.00069-23

**Published:** 2023-04-26

**Authors:** Ainsley R. Chapman, Jillian M. Wright, Nicole A. Kaiser, Peter M. Jones, Erin M. Driver, Rolf U. Halden, Arvind Varsani, Matthew Scotch, Temitope O. C. Faleye

**Affiliations:** a Biodesign Institute Center for Environmental Health Engineering, Arizona State University, Tempe, Arizona, USA; b University of Arizona College of Medicine-Phoenix, Phoenix, Arizona, USA; c College of Health Solutions, Arizona State University, Tempe, Arizona, USA; d School of Sustainable Engineering and the Built Environment, Arizona State University, Tempe, Arizona, USA; e OneWaterOneHealth, Arizona State University Foundation, Tempe, Arizona, USA; f Biodesign Center for Fundamental and Applied Microbiomics, Center for Evolution and Medicine, School of Life Sciences, Arizona State University, Tempe, Arizona, USA; Queens College Department of Biology

## Abstract

We describe the genome (4,696 nucleotides [GC content, 56%; coverage, 3,641×) of MAZ-Nov-2020, a microvirus identified from municipal wastewater in Maricopa County, Arizona, USA, in November 2020. The MAZ-Nov-2020 genome encodes major capsid protein, endolysin, replication initiator protein, and two hypothetical proteins, one of which was predicted to likely be a membrane-associated multiheme cytochrome *c*.

## ANNOUNCEMENT

Microviruses (family *Microviridae*) are small single-stranded DNA (ssDNA) viruses that are widespread in diverse environments (1). They have icosahedral capsids and circular 4- to 6.5-kb ssDNA genomes. Despite their abundance, our understanding of their diversity, evolutionary dynamics, and abundance is limited (2).

The MAZ-Nov-2020 microvirus genome was serendipitously identified from a 1,000-fold (2 L to 2 mL) concentrated (using an ultrafiltration device with a molecular weight cutoff value of 10,000) wastewater (WW) sample that had been collected in Maricopa County, Arizona, USA, in November 2020 as part of a canine picornavirus WW surveillance study (3). The concentrate was subjected to nucleic acid extraction (QIAamp minikit), pan-canine picornavirus reverse transcription (RT)-PCR (3), library preparation (KAPA HyperPlus library kit), and Illumina sequencing (MiSeq system with 2 × 250-bp paired-end reads). Raw reads were trimmed and *de novo* assembled, contigs were identified, and the depth of coverage was ascertained using Trimmomatic v0.36, metaSPades v3.15.3, a BLASTn search of the GenBank database, and Bowtie2 v2.3.2, respectively, with default parameters on the KBase platform (4). The microvirus genome was annotated using the autoannotate function in DNA Master (5) and GeneMarkS (6). Functional annotation of predicted open reading frames (ORFs) was performed using BLASTp, HMMER, and I-TASSER (7–9). For phylogenetic analysis, all 75 major capsid protein (MCP) GenBank protein hits were downloaded and aligned using MUSCLE in MEGA11 (10), and a maximum likelihood (ML) tree was constructed with 1,000 bootstrap replicates using IQTree v1.6.12 (11). Pairwise identities were determined using SDT v1.2 (12), and the tree was visualized and annotated using iTOL v6 (13). PCR confirmation of microvirus MAZ-Nov-2020 in the sample was performed using primers Rhph-F (5′-GCCTCGGTTCTGAATTCTGCGGGGTTTACTTCGG-3′) and Rhph-R (5′-TTAAGGCGCGGAGCCTTGGCAACCTTCATTCC-3′) with Phusion Plus Green master mix and the following reaction conditions: 94°C for 3 min, 40 cycles of 94°C for 30 s, 55°C for 30 s, and 68°C for 6 min, and finally 68°C for 10 min.

**FIG 1:**
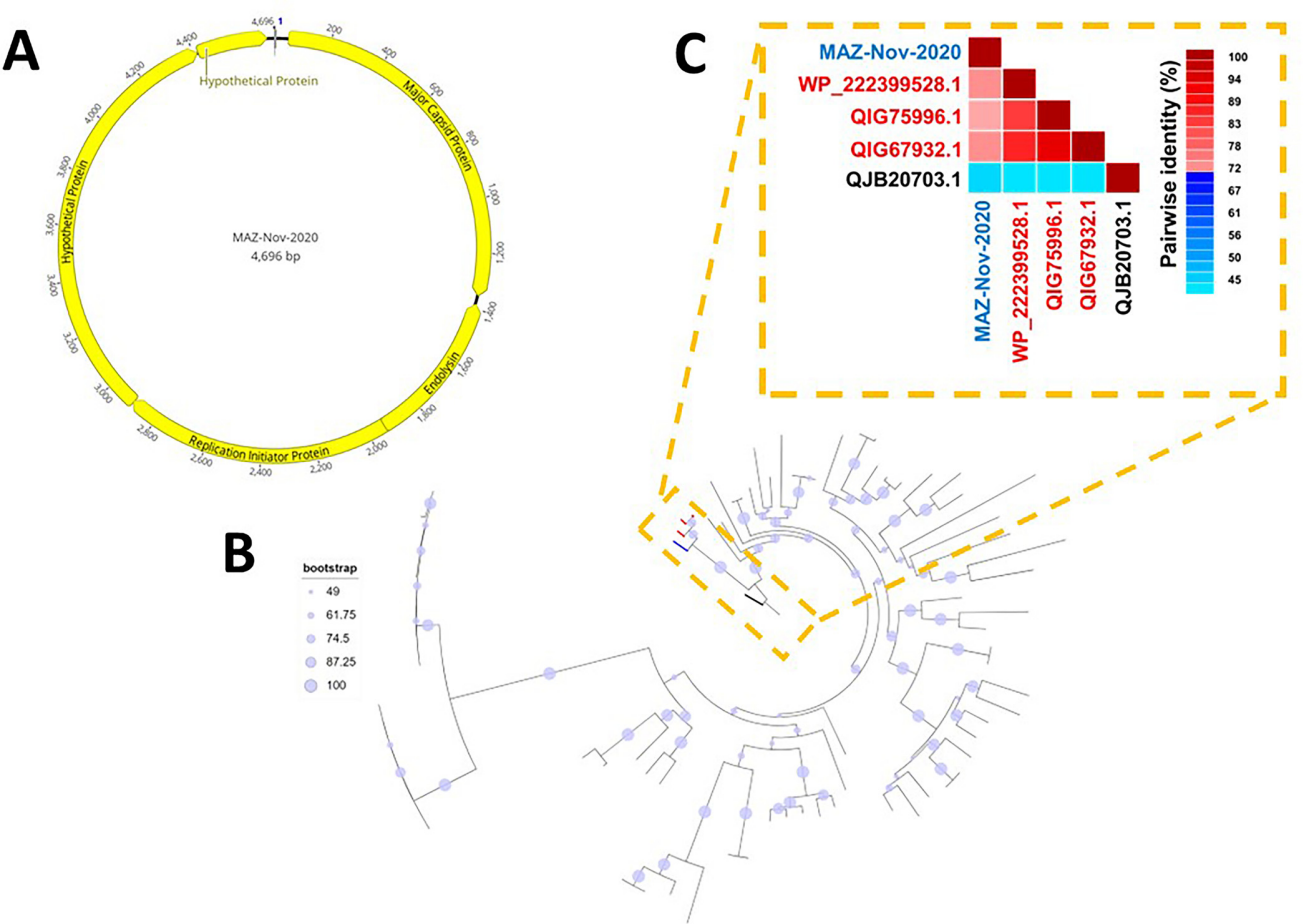
(A) Genome map of MAZ-Nov-2020. (B) ML phylogenetic tree of 75 GenBank hits and the MCP of MAZ-Nov-2020. The tree was constructed using IQTree v1.6.12 with the best-fit model according to the Bayesian information criterion (BIC) with LG+F+I+G4. (C) Pairwise identity analysis of sequences that clustered (red) with MZ-Nov-2020 (blue) in panel B, as well as the outgroup QJB20703.1 (black).

MAZ-Nov-2020 (4,696 nucleotides [nt] [GC content, 56%; coverage, 3,641×]) was assembled from 6.54% of the trimmed raw reads (93,084/1,423,442 reads) and circularized via terminal redundancy. A BLASTn search of MAZ-Nov-2020 showed that it is most similar to GenBank accession number MN988485 (query coverage, 40%; identity, 69.61%), a member of putatively named subfamily *Amoyvirinae* (2). Autoannotation of MAZ-Nov-2020 predicted 5 hypothetical ORFs (Table 1 and Fig. 1A), 3 of which were predicted (with BLASTp, I-TASSER, and HMMER) to encode MCP, endolysin, and replication initiator protein (Table 1 and Fig. 1A). I-TASSER predicted that ORF4 is likely a membrane-associated multiheme cytochrome *c* and ORF5 is also likely a membrane-associated protein (Table 1). BLASTp showed MAZ-Nov-2020-MCP to be most similar to GenBank accession number WP_222399528 (Rhizobium leguminosarum) and *Rhizobium* phage MCPs QIG67932 (GenBank accession number MN988485) and QIG75996 (GenBank accession number MN988550), with pairwise identities of 72.24%, 72.24%, and 70.59%, respectively. These suggest members of this clade have both lytic and lysogenic phases in their life cycle (2) and might be affecting biogeochemical processes such as nitrogen fixation. ML trees for all 75 MCP BLASTp hits and the pairwise identities suggested that MAZ-Nov-2020 is a *Rhizobium* phage-like virus (Fig. 1B and C).

**TABLE 1:** Properties of ORFs predicted in MAZ-Nov-2020

Parameter	Data for ORF:				
	1	2	3	4	5
ORF positions (start to stop)	50–1351	1383–1937	1930–2904	2901–4415	4417–4671
ORF length (nt)	1,302	555	975	1,515	255
Encoded protein length (amino acids)	434	185	325	505	85
Encoded protein start/stop codons	ATG/TAA	ATG/TGA	GTG/TGA	GTG/TGA	ATG/TGA
Reading frame	RF2	RF5	RF1	RF3	RF1
Direction	Forward	Reverse	Forward	Forward	Forward
BLASTp results					
GenBank accession no.	WP_222399528, QIG67932	QIG67933	WP-222399530	QIG75994	No BLASTp results
Alignment (%)	99.8, 99.8	94	92.8	60.3	
Identity (%)	72.24, 72.24	44.68	63.31	63.27	
Function	Hypothetical, putative MCP	Endolysin	Replication initiator protein	Hypothetical	
HMMER results					
Accession number	A0A4P8WAA5_9VIRU	A0A0F2J6V4_9BACT	A0A2H4PJ67_9VIRU	A0A4V1FVZ2_9VIRU	No significant match
E value	1.8e−76	4.2e−11	8.9e−35	1.5e−4	
Function	MCP	Endolysin	Replication initiator protein	Uncharacterized protein	
I-TASSER results					
PDB code	1KVP	2VO9	7LCC	4QO5	4HYC
TM-Align score (%)	83.5	68.3	80	87.5	55.7
RMSD^*a*^	2.53	1.83	3.37	1.33	3.29
Identity (%)	13	19	14.3	9.5	12.2
Coverage (%)	89.6	73.9	92.6	89.7	87.1
Function	MCP	Endolysin	DNA transposase with ssDNA-specific HUH endonuclease domain	Membrane-associated octaheme cytochrome *c*	Presenilin family intramembrane protease
Summary	MCP	Endolysin	Replication initiator protein	Membrane-associated multiheme cytochrome *c*	Intramembrane protein

a RMSD, root mean square deviation.

The microvirus MAZ-Nov-2020 genome encodes an endolysin with antibacterial potential (14), as well as a previously undescribed putative multiheme cytochrome *c*-like protein (Table 1) with potential application in bionanoelectronic devices (15). Expanding our knowledge of these viruses and their unexplored encoded protein functions will further our understanding of their diversity, evolutionary dynamics, and abundance.

### Data availability.

The mapped reads and the genome described in this study have been deposited in the SRA and GenBank under BioProject accession number PRJNA918393 and GenBank accession number OQ184949, respectively.
